# Promotive role of IRF7 in ferroptosis of colonic epithelial cells in ulcerative colitis by the miR-375-3p/SLC11A2 axis

**DOI:** 10.17305/bjbms.2022.8081

**Published:** 2023-05-01

**Authors:** Zepeng Chen, Qinglong Gu, Ruichao Chen

**Affiliations:** 1 Department of Anorectal Surgery, The Affiliated Hospital of Nanjing University of Chinese Medicine, Nanjing, China; 2 First Clinical Medical College, Nanjing University of Chinese Medicine, Nanjing, China; 3 Department of Anorectal Surgery, Xuzhou City Hospital of TCM, Xuzhou, China

**Keywords:** Ulcerative colitis (UC), ferroptosis, interferon regulatory factor 7 (IRF7), SLC11A2, miR-375-3p, glutathione peroxidase 4 (GPX4)

## Abstract

Ferroptosis is implicated in the progression of ulcerative colitis (UC), and interferon regulatory factor 7 (IRF7) contributes to cell death. This study probed the mechanism of IRF7 in ferroptosis of colonic epithelial cells (ECs) in mice with dextran sodium sulfate (DSS)-induced UC. The UC mouse model and the in vitro ferroptosis model were, respectively, established by DSS feeding and the treatment with FIN56 (a ferroptosis inducer). Results of quantitative real-time polymerase chain reaction and western blotting revealed the upregulation of IRF7 and solute carrier family 11 member 2 (SLC11A2/NRAMP2/DMT1) and the downregulation of microRNA (miR)-375-3p in DSS-treated mice and FIN56-treated ECs. Silencing of IRF7 improved the symptoms of UC in DSS-induced mice and decreased the levels of tumor necrosis factor α, interleukin 6, monocyte chemoattractant protein 1, and interleukin 1β, reactive oxygen species, iron ions, lipid peroxidation, and increased glutathione and glutathione peroxidase 4. Chromatin immunoprecipitation and dual-luciferase assays showed that binding of IRF7 to the miR-375-3p promoter inhibited miR-375-3p expression, and miR-375-3p suppressed SLC11A2 transcription. The rescue experiments revealed that knockdown of miR-375-3p neutralized the role of silencing IRF7 in alleviating ferroptosis of colonic ECs. Overall, IRF7 upregulated SLC11A2 transcription by inhibiting miR-375-3p expression, thereby prompting ferroptosis of colonic ECs and UC progression in DSS-treated mice.

## Introduction

Ulcerative colitis (UC), first described by Samuel Wilks in 1859, is defined as a long-lasting and immune-mediated inflammatory bowel disorder (IBD) featured by persistent and diffusive intestinal inflammation, which is confined to the colonic mucosa and spreads from the rectum to the proximal areas [1]. Generally, potential antigens in response to the environmental factors and intestinal microbiota initiate multifactorial conditions to render maladjusted immune and inflammatory cascades. Consequent release of proinflammatory cytokines directly contribute to epithelial cell (EC) and tissue dysfunction and intestinal barrier impairment [2, 3]. Ferroptosis, first discovered by Dixon et al. in 2012, is a distinctive type of cell death that is associated with dysregulated deposition of iron and iron-dependent lipid peroxidation [4, 5]. Ferroptosis initiates damage-associated molecular patterns to trigger inflammatory responses and oxidative stress, therefore facilitating numerous diseases, such as cardiovascular disease and acute kidney injury [6–8]. Ferroptosis plays an emerging role in prompting the progression of IBD [9] and accelerates intestinal EC death in UC under the regulation of nuclear factor κ Bp65 subunit (NF-κBp65)-mediated endoplasmic reticulum stress signaling [10]. Besides, the suppression of ferroptosis in UC is associated with the inactivation of the nuclear factor erythroid 2-like 2/heme oxygenase-1 [11]. Moreover, the activation of glutathione peroxidase 4 (GPX4), a key marker for modulating ferroptosis by decreasing lipid peroxidation, has been reported to reduce EC injury and ameliorate UC [12, 13]. In this work, the precise mechanism of ferroptosis of colonic ECs is further explored.

Interferon regulatory factors (IRFs), a set of transcription factors, are composed of nine members and responsible for the control of interferons (IFNs)-mediated responses, and a variety of immune activities, including anti-viral and proinflammatory responses [14, 15]. As the key controller of type I IFN, the strict regulation of the expression and activation of IRF7 is crucial to manage the appropriate production of type I IFN to prevent diseases caused by dysregulated type I IFN [16, 17]. Moreover, IRF7 upregulation in intestinal dendritic cells (DCs) worsens IBD progression [18]. Nevertheless, IRF7-mediated regulation of ferroptosis of colonic ECs in UC remains undiscovered.

IRF7, as a transcription factor, functions in disease by targeting downstream genes [19]. microRNAs (miRs), a subcategory of noncoding RNAs, affect gene expression at the post-transcriptional level by interacting with the target messenger RNAs (mRNAs) and act as regulators of cellular processes and animal development [20]. Aberrantly expressed miRs are implicated with the impaired function of mucosal immunity and the increased production of proinflammatory cytokines in IBD [21]. Besides, miRs exert anti-ferroptotic or proferroptotic activities by modulating their target genes [22]. Furthermore, miR-375-3p downregulation is associated with the severity of pediatric UC [23]. Additionally, the JASPAR database revealed that the binding of miR-375-3p to solute carrier family 11 member 2 (SLC11A2/NRAMP2/DMT1). SLC11A2 is a major transmembrane iron transporter for supporting iron absorption and metabolism in various tissues, including the bowel [24, 25]. SLC11A2-mediated release of iron modulates the recycling of transferrin and transferrin receptors and abnormal transferrin receptors expression results in ferroptosis [26]. Besides, ferroptosis induces the excessive iron uptake by SLC11A2 [27], and in turn, SLC11A2 overexpression leads to glutathione (GSH) decrease and presents propensity to ferroptosis [28, 29]. Based on the currently available evidence, we attempted to address our speculation whether IRF7 had an impact on ferroptosis of colonic ECs in UC by manipulating the miR-375-3p/SLC11A2 axis so as to provide a novel insight into the therapeutic approach to UC.

## Materials and methods

### Animal modeling and treatment

C57BL/6J male mice (age 6–8 weeks; Beijing Vital River Laboratory Animal Technology Co., Ltd., Beijing, China; License number: SCXK (Beijing) 2019-0009) were housed at a temperature of 25 ^∘^C and a humidity of 45%–55%, under 12-h light/dark cycles, with free access to food and water. This animal scheme was authorized by the Animal Ethics Committee of The Affiliated Hospital of Nanjing University of Chinese Medicine and conducted in accordance with the Guide for the Care and Use of Laboratory Animals [30].

Mice were given drinking water containing 3% dextran sodium sulfate (DSS; Sigma, St. Louis, MO, USA) for 7 consecutive days to induce the UC mouse model, while mice in the sham group were given DSS-free drinking water. Adenovirus-packaged vectors containing the sh-IRF7 sequences and the corresponding control (sh-NC) were obtained from GenePharma (Shanghai, China) and were injected (1 × 10^9^ viral genomes/µL) into mice via tail veins 2 weeks and 1 week before DSS induction. A total of 72 experimental mice were divided into the sham group, the DSS group, the DSS + sh-NC group, and the DSS + sh-IRF7 group, with 18 mice in each group.

**Figure 1.**
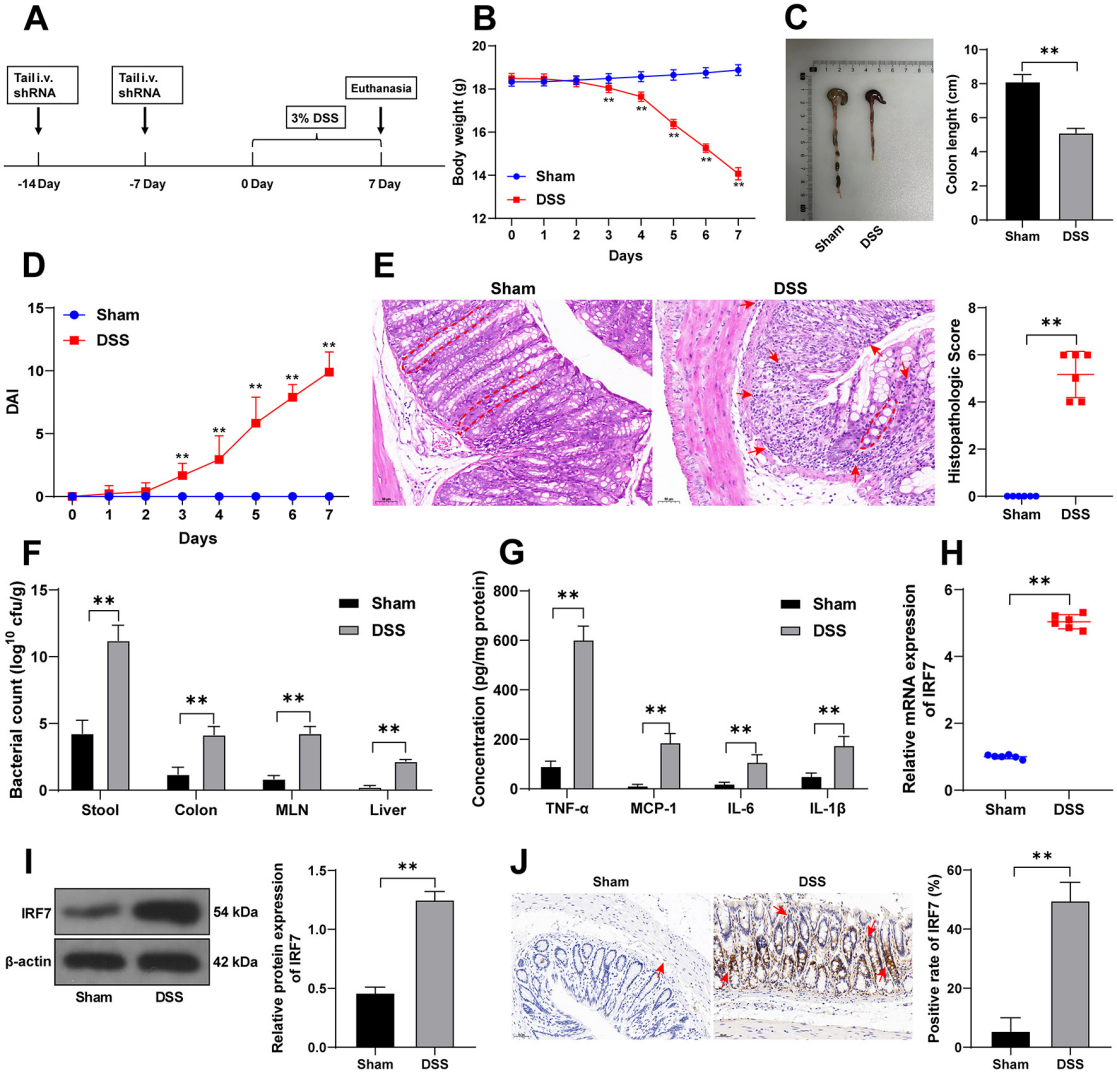
**IRF7 is robustly expressed in DSS-treated mice.** (A) Mice were given drinking water containing 3% DSS for 7 days to establish the mouse UC model, and mice in the sham group were given DSS-free drinking water; (B) Changes in body weight of mice, *n* ═ 18; (C) The length of the colons of mice, *n* ═ 18; (D) The DAI scores, *n* ═ 18; (E) H&E staining and the histopathologic scores, and the arrows and dotted lines indicated the areas with EC damage, crypt distortion, and massive inflammatory cell infiltration, *n* ═ 6; (F) Bacteria number in stool, colonic, MLN, and liver tissues of mice, *n* ═ 6; (G) The levels of TNF-α, MCP-1, IL-6, and IL-1β in colonic tissues were detected using ELISA, *n* ═ 6; (H) and (I) The expression levels of IRF7 in colonic tissues were detected using qRT-PCR and western blotting, *n* ═ 6; (J) The positive expressions of IRF7 in colonic tissues were observed using the IHC staining, and arrows indicated representative positive expression, *n* ═ 6. Comparisons in panels B, D, F, and G were assessed by two-way ANOVA; pairwise comparisons after ANOVA analyses were assessed by Tukey’s multiple comparison test; comparisons in panels C, E, H, I, and J were assessed by the *t*-test; **p < 0.01. IRF7: Interferon regulatory factor 7; DSS: Dextran sodium sulfate; DAI: Disease activity index; H&E: Hematoxylin-eosin; TNF-α: Tumor necrosis factor α; MCP-1: Monocyte chemoattractant protein 1; IL: Interleukin; EC: Epithelial cells; ELISA: Enzyme-linked immunosorbent assay; qRT-PCR: Quantitative real-time polymerase chain reaction; IHC: Immunohistochemistry; MLN: Mesenteric lymph nodes; UC: Ulcerative colitis; ANOVA: Analysis of variance.

Mouse weight was observed and recorded daily, and mice were euthanized (intraperitoneal injection of 200 mg/kg pentobarbital sodium) on day 7 of treatment to obtain mouse colonic tissues (Figure 1A). After the measurement of the length of the mouse colons, the follow-up tests were performed. Then, six mice in each group were randomly chosen for the histopathological staining and examination, with other six mice in each group for the bacteria culture, and the remaining six mice in each group for the homogenization processing.

### Disease activity index score

The body weight, stool consistency, and stool blood volume of mice were measured based on a previously reported grading system [31] to assess the severity of colitis. The scoring criteria for weight loss were as follows: point 0 ═ none; point 1 ═ 1%–5%; point 2 ═ 5%–10%; point 3 ═ 10%–20%; point 4 > 20%. The scoring criteria for diarrhea were as follows: point 0 ═ normal; point 2 ═ loose stool; point 4 ═ watery diarrhea. The scoring criteria for stool blood volume were as follows: point 0 ═ normal; point 2 ═ hyporrhea; point 4 ═ hemorrhea. These comprised the disease activity index (DAI) score for determining disease severity.

### Hematoxylin-eosin staining

The obtained colonic tissues were fixed with 4% paraformaldehyde, paraffin-embedded, sliced into 5 µm-thick sections, and subjected to hematoxylin-eosin (H&E) staining. With reference to the scoring criteria [32], pathologists were asked to score histological changes using the blind method. The histological changes included inflammatory cell infiltration (point: 0–3) and tissue damage (point: 0–3).

### Immunohistochemistry

After being dewaxed and hydrated, paraffin sections were incubated with 3% H_2_O_2_ for 20 min to block the activity of endogenous peroxidase. Thereafter, sections were blocked with 10% fetal calf serum for 1 h, and incubated with anti-GPX4 antibody (1:50, ab125066, Abcam, Cambridge, MA, USA) and IRF7 (1:100, PA5-114526, Invitrogen, Carlsbad, CA, USA) overnight at 4 ^∘^C and with secondary antibody IgG (1:200, ab6721, Abcam) for 30 min at room temperature. Slides were stained with 3,3’-dimethylbenzidine, dehydrated, sealed with neutral glue, and observed using a microscope (Olympus CKX51, Olympus, Tokyo, Japan).

### Bacterial culture

The samples of stool, colons, mesenteric lymph nodes (MLN), and liver tissues from 6 mice randomly selected from each group were collected in 5 mL of 3% thioglycolate reagent and then homogenized. The suspensions of different dilutions were coated on blood agar and brain–heart–infusion agar and incubated for 48 h at 37 ^∘^C. Then, colony formation assay was performed to count the number of bacterial colonies.

### Enzyme-linked immunosorbent assay

Parts of the colonic tissues were mechanically homogenized in phosphate-buffered saline (PBS) containing of a mixture of NP-40 lysis buffer (1%) and complete protease inhibitors. Following the manufacturer’s instructions (R&D Systems, Minneapolis, MN, USA), levels of tumor necrosis factor α (TNF-α) (MTA00B), interleukin 6 (IL-6) (M6000B), IL-1β (MLB00C), and monocyte chemoattractant protein 1 (MJE00B) in the colonic homogenates were examined by the commercial enzyme-linked immunosorbent assay (ELISA) kits.

### Cell isolation and culture

The colons of mice in each group were dissected, rinsed with PBS, and cut into small pieces, and these pieces were shaken in Hank’s balanced salt solution containing 5 mM ethylene diamine tetraacetic acid and 0.5 mM dithiothreitol for 30 min. Cells in the supernatant were filtered using a 70-mm cell filter and washed twice. The enrichment of colonic ECs was measured by the percentage of stained cells positive for cytokeratin 18 (an EC-specific marker, 1:1000, ab181597, Abcam), which revealed that 85%–90% of the isolated cells stained positive for cytokeratin 18. After identification, mouse colonic ECs were loaded in an Eagle’s minimum essential medium added with 10% fetal calf serum, 2 mM L-glutamine, 100 IU penicillin, and 100 mg/mL streptomycin at 37 ^∘^C with 5% CO_2_.

### Cell transfection and treatment

The colonic ECs of mice in the control group were seeded in 6-well plates, infected with adenovirus-packaged sh-IRF7, and screened using puromycin, and colonic ECs with stably silenced IRF7 were obtained after two weeks. pcDNA3.1-IRF7 (oe-IRF7), miR-375-3p inhibitor (miR-inhi), miR-375-3p mimic, and the controls (oe-NC, inhi-NC, and mimic-NC) were brought from GenePharma and transfected into colonic ECs using Lipofectamine 3000 (Invitrogen) based on the manufacturer’s instructions. The transfection efficiency was examined using quantitative real-time polymerase chain reaction (qRT-PCR) and western blotting 48 h after transfection. Cell medium after transfection was added with the ferroptosis inducer FIN56 (5 µM, HY-103087, MedChemExpress, NJ, USA) to establish the in vitro ferroptosis cell model, and the follow-up assays were conducted 12 h after the model establishment.

### Cell counting kit 8 assay

Cell viability of mouse colonic ECs was measured with cell counting kit 8 (CCK-8) (Dojindo Laboratories, Shanghai, China). The colonic ECs (n ═ 3× 10^3^ cells) of different groups were seeded in 96-well plates, and each well was added with 200 µL of medium supplemented with 10 µL CCK-8 reagent and incubated under the dark conditions for 2 h. The optical density (OD) at 450 nm was detected by a microplate reader, and the cell viability was measured, with the results from the control group as the standard.

### Detection of iron levels

Protein of tissues or cells was prepared using an iron assay kit (ab83366, Abcam) as described in the instructions. Wells were supplemented with 100 µL standard diluent and samples, and 5 µL of iron reducing agent was added to each well. Thereafter, the each well containing standard iron and samples was added with 100 µL Iron Probe, mixed, and incubated at 37 ^∘^C for 1 h under the dark conditions. OD (at 593 nm) was detected using a microplate reader.

### Determination of reactive oxygen species and glutathione levels

Protein samples were yielded from tissues and cells to determine GSH levels using a reduced GSH detection kit (A006-2, Nanjing Jiancheng Bioengineering Institute, Nanjing, Jiangsu, China). Following the producer’s instructions, reactive oxygen species (ROS) levels in tissues and cells were examined using the fluorescent probe DCFH-DA (S0033S, Beyotime, Shanghai, China). The lysed tissues and cells were added with DCFH-DA and incubated at 37 ^∘^C for 20 min and washed with PBS 3 times. The changes in the fluorescent intensity at a wavelength of 500/530 nm were determined using a fluorescent microplate reader (Bio-Rad, Hercules, CA, USA), and the results were compared to the fluorescence intensity of the control group.

### Assessment of lipid peroxidation

To detect cell lipid peroxidation, colonic ECs were seeded in 8-well plates and incubated with C11-BODIPY^581/591^ (2 µM) in growth media for 30 min at 37 ^∘^C under the dark conditions. The double wavelength excitation (488 and 568 nm) and detection (emission bandpass filters 530/30 and 590/30) were used to develop the green and red fluorescence of C11-BODIPY^581/591^. Images were captured using a TCSNT confocal laser scanning system (Leica, Wetzlar, Hessian, Germany).

### Quantitative real-time polymerase chain reaction

The total RNA in mouse colonic tissues and ECs was extracted using the TRIzol reagent (Invitrogen) and was reverse-transcribed into the complementary DNA using the SuperScript IV reverse transcriptase (ThermoFisher Scientific, Waltham, MA, USA). qRT-PCR reactions were performed on Taqman general PCR Master mix II (ThermoFisher Scientific), and the relative gene expression was calculated using the 2^−ΔΔCt^ method [33] with GAPDH or U6 [34] as the internal references. The used primer sequences are shown in Table 1.

**Table 1:** Polymerase chain reaction primers

**Genes**	**Sequences (**5’-3’**)**
*IRF7*	F: GTGAGGGGGGTCCAGCGAGTGCT
	R: CTCCACTAGGTGGCCACCTCCCTC
*SLC11A2*	F: GGATCCTAAAGAAAAGATGCCAG
	R: CTAGGTAGGCAATGCTCATAAGA
*GAPDH*	F: ATGCTGCCCTTACCCCGGGGT
	R: TTACTCCTTGGAGGCCATGTAG
*U6*	F: ATGGCGGACGACGTAGATCA
	R: TCAGCCAACTCTCAATGGAG
miR-375-3p	F: GCCGAGTTTGTTCGTTCGGC
	R: CTCAACTGGTGTCGTGGA
miR-375-3p promoter	F: GCTGATCTTTGCAACAGTGTAACT
	R: GATTAAAGGCTTCCTTGCTTGCGC

IRF7: Interferon regulatory factor 7; GAPDH: Glyceraldehyde 3-phosphate dehydrogenase; miR: MicroRNA.

### Western blotting

Mouse colonic tissues and ECs were lysed using the radioimmunoprecipitation assay (RIPA) lysis buffer supplemented with a mixture of protease inhibitors to obtain protein samples, and samples were centrifugated and the protein concentration was examined by a bicinchoninic acid kit (P0011, Beyotime). Equal amounts of proteins were received sodium dodecyl sulfate–polyacrylamide gel electrophoresis and transferred onto polyvinylidene fluoride membranes, and after blockade with defatted milk, the membranes were incubated with primary antibodies against GPX4 (1:1000, ab125066, Abcam), IRF7 (1:1000, ab288440, Abcam), SLC11A2 (1:1000, ab55735, Abcam), and β-actin (1:5000, ab6276, Abcam) and with appropriate amount of horse radish peroxidase-conjugated secondary antibody IgG (1:1000, ab6721, Abcam). Protein signals were visualized using the enhanced-chemiluminescence reagent (P0018, Beyotime).

### Chromatin-immunoprecipitation assay

The chromatin-immunoprecipitation (Ch-IP) assay was conducted using the Simple Ch-IP Enzymatic Chromatin IP kit (Cell Signaling Technology, Danvers, MA, USA). The colonic ECs were lysed using the RIPA buffer (Sigma, St. Louis, MO, USA), fixed with 1% formaldehyde to crosslink DNA and proteins, and ultrasonically processed to fragment DNA. Immunoprecipitation was performed on chromatin using anti-IRF7 (SC0617, Novus Biologicals, Littleton, CO, USA) or anti-IgG (the negative control; ab6721, Abcam), and chromatin was purified using a fragment DNA purification kit (iNtRON Biotechnology, Seongnam, Gyeonggi-do, South Korea) and examined using qRT-PCR. The primer of the miR-375-3p promoter is shown in Table 1.

### Dual-luciferase assay

First, the binding sites of IRF7 and the miR-375-3p promoter sequence were predicted by the database JASPAR (http://jaspar.genereg.net/) [35]. Then, the miR-375-3p promoter sequence containing the binding sites of IRF7 was inserted into the luciferase reporter vector (Promega, Madison, WI, USA) to construct the wild-type plasmid of the miR-375-3p promoter (miR-375-3p-WT). As well, the sequence containing the mutant binding sites was inserted into the luciferase reporter vector to construct the mutant-type plasmid of the miR-375-3p promoter (miR-375-3p-MUT). In the same manner, according to the binding sites of miR-375-3p and the SLC11A2 3’UTR sequence predicted by the database Targetscan (https://www.targetscan.org/vert/_71/) [36], the SLC11A2-WT and SLC11A2-MUT containing the binding sites were constructed, respectively. Afterward, these constructed vectors containing the miR-375-3p promoter sequence were co-transfected with pcDNA3.1-IRF7 (oe-IRF7) or empty-vector (oe-NC) into colonic ECs using Lipofectamine 3000, and the vectors containing the SLC11A2 3’UTR sequences were co-transfected into colonic ECs with miR-375-3p mimic or its control (mimic-NC). Following the producer’s instructions, the luciferase activity was examined by a dual-luciferase reporter assay kit (Promega) 48 h after transfection.

### Ethical statement

This animal scheme was authorized by the Animal Ethics Committee of The Affiliated Hospital of Nanjing University of Chinese Medicine (approval number 2022-NJ043) and conducted in accordance with the Guide for the Care and Use of Laboratory Animals [30].

### Statistical analysis

Statistical analysis was conducted by SPSS 21.0 (SPSS Inc., Chicago, IL, USA). Data were normally distributed as tested by Kolmogorov–Smirnov and expressed as mean ± standard deviation. The *t*-test was applied to evaluate statistical differences between the two groups. One-way or two-way analysis of variance (ANOVA) was applied to evaluate statistical differences among multi-groups. After ANOVA, Tukey’s multiple comparison test was applied for the post test. *p* value was attained from the two-sided test, and *p* < 0.05 represented statistical significance and *p* < 0.01 represented highly statistical significance.

## Results

### IRF7 is robustly expressed in DSS-treated mice

The mouse UC model was established using DSS (Figure 1A). The body weight of DSS-treated mice decreased continuously within seven days (*p* < 0.01, Figure 1B) and the length of the colons shrank (*p* < 0.01, Figure 1C). The DAI scores of DSS-treated mice rose rapidly (*p* < 0.01, Figure 1D) and H&E staining showed that the colonic tissues of DSS-treated mice presented notable EC damage, crypt distortion, and massive inflammatory cell infiltration (*p* < 0.01, Figure 1E). Meanwhile, bacterial colonies in stool, MLN, colons, and liver tissues of mice in the DSS group were increased (*p* < 0.01, Figure 1F), and the levels of TNF-α, monocyte chemoattractant protein 1, IL-6, and IL-1β in the colonic tissues of DSS-treated mice were elevated (*p* < 0.01, Figure 1G). Lastly, IRF7 expression levels in mouse colonic tissues were detected, and IRF7 expression levels were increased in the DSS group (*p* < 0.01, Figure 1H– 1J). These observations indicated the successful establishment of the UC mouse model and that IRF7 was highly expressed in mice with DSS-induced UC.

### IRF7 knockdown mitigates UC symptoms in DSS-treated mice

**Figure 2.**
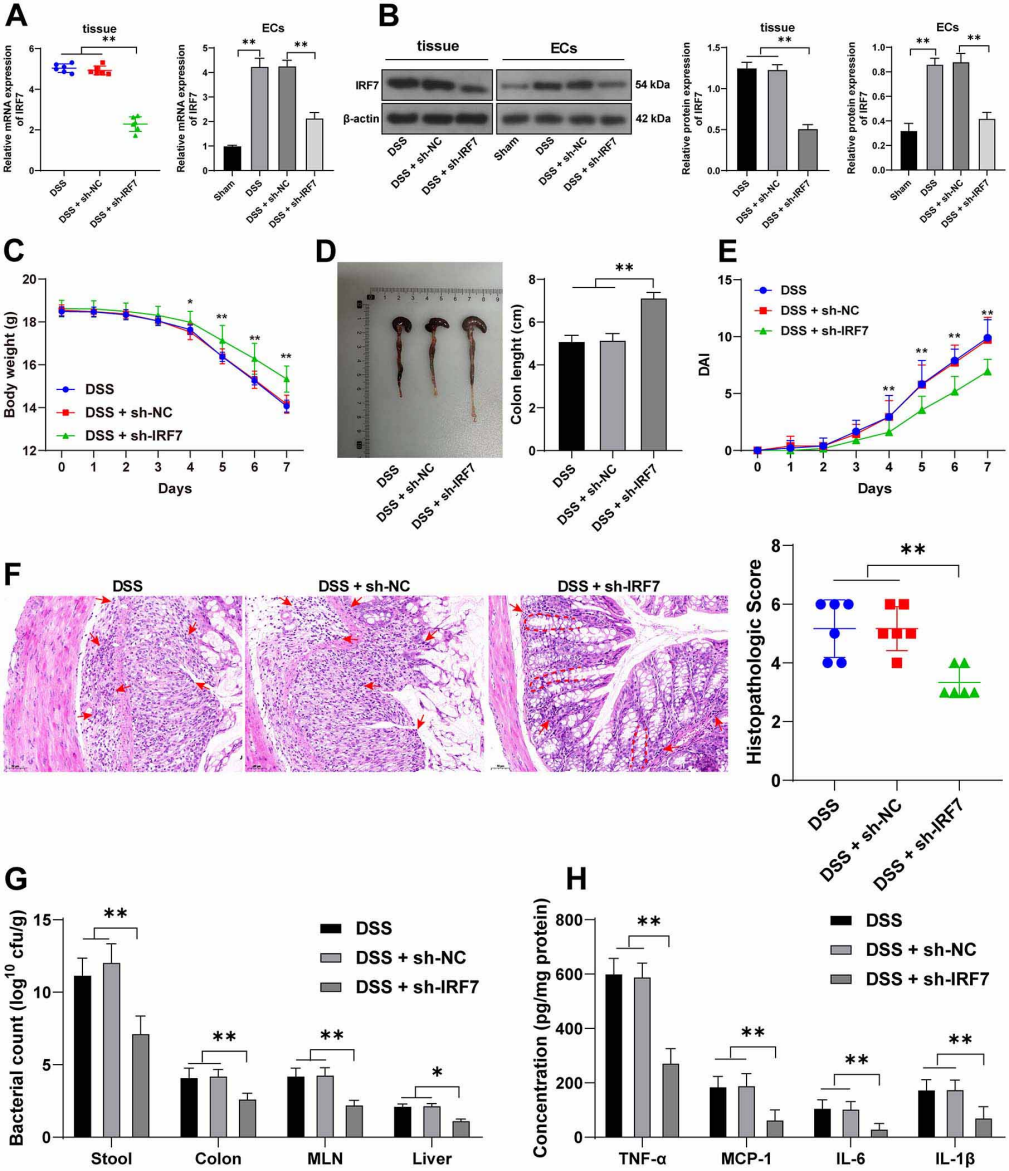
**IRF7 knockdown mitigates UC symptoms in DSS-treated mice.** Mice were injected with adenovirus-packaged sh-IRF7 via tail veins, with adenovirus-packaged sh-NC as the control. (A) and (B) The expression levels of IRF7 in colonic tissues and ECs were, respectively, detected using qRT-PCR and western blotting, *n* ═ 6; (C) Changes in body weight of mice over 7 days, *n* ═ 18; (D) The length of the colons of mice, *n* ═ 18; (E) The DAI scores, *n* ═ 18; (F) H&E staining and the histopathologic scores, and the arrows and dotted lines indicated the areas with EC damage, crypt distortion, and massive inflammatory cell infiltration, *n* ═ 6; (G) Bacteria number in stool, colons, MLN, and liver tissues of mice, *n* ═ 6; (H) The levels of TNF-α, MCP-1, IL-6, and IL-1β in colonic tissues were detected using ELISA, *n* ═ 6. Comparisons in panels A, B, D, and F were assessed by one-way ANOVA; comparisons in panels C, E, G, and H were assessed by two-way ANOVA; pairwise comparisons after ANOVA analyses were assessed by Tukey’s multiple comparison test; *p < 0.05 and **p < 0.01. IRF7: Interferon regulatory factor 7; UC: Ulcerative colitis; DSS: Dextran sodium sulfate; DAI: Disease activity index; H&E: Hematoxylin-eosin; TNF-α: Tumor necrosis factor α; MCP-1: Monocyte chemoattractant protein 1; IL: Interleukin; EC: Epithelial cells; ELISA: Enzyme-linked immunosorbent assay; qRT-PCR: Quantitative real-time polymerase chain reaction; IHC: Immunohistochemistry; MLN: Mesenteric lymph nodes; ANOVA: Analysis of variance.

To explore the role of IRF7 in UC, adenovirus-packaged sh-IRF7 was used to decrease IRF7 expression levels in mice, and detections showed that IRF7 expression levels were decreased in colonic tissues and ECs (*p* < 0.01, Figures 1A and 2A and 2B). After successful knockdown of IRF7, DSS-treated mice showed significant improvement in UC symptoms, as reflected by increased body weight, increased colon length, and decreased DAI scores (*p* < 0.05, Figure 2C–2E). Besides, IRF7 knockdown alleviated histopathological damages (*p* < 0.01, Figure 2F), reduced bacterial colonies in stool, MLN, colons, and liver tissues (*p* < 0.05, Figure 2G), and reduced the levels of proinflammatory cytokines (*p* < 0.01, Figure 2H). These results suggest that IRF7 knockdown ameliorated DSS-induced UC in mice.

### IRF7 knockdown limits DSS-induced ferroptosis

**Figure 3.**
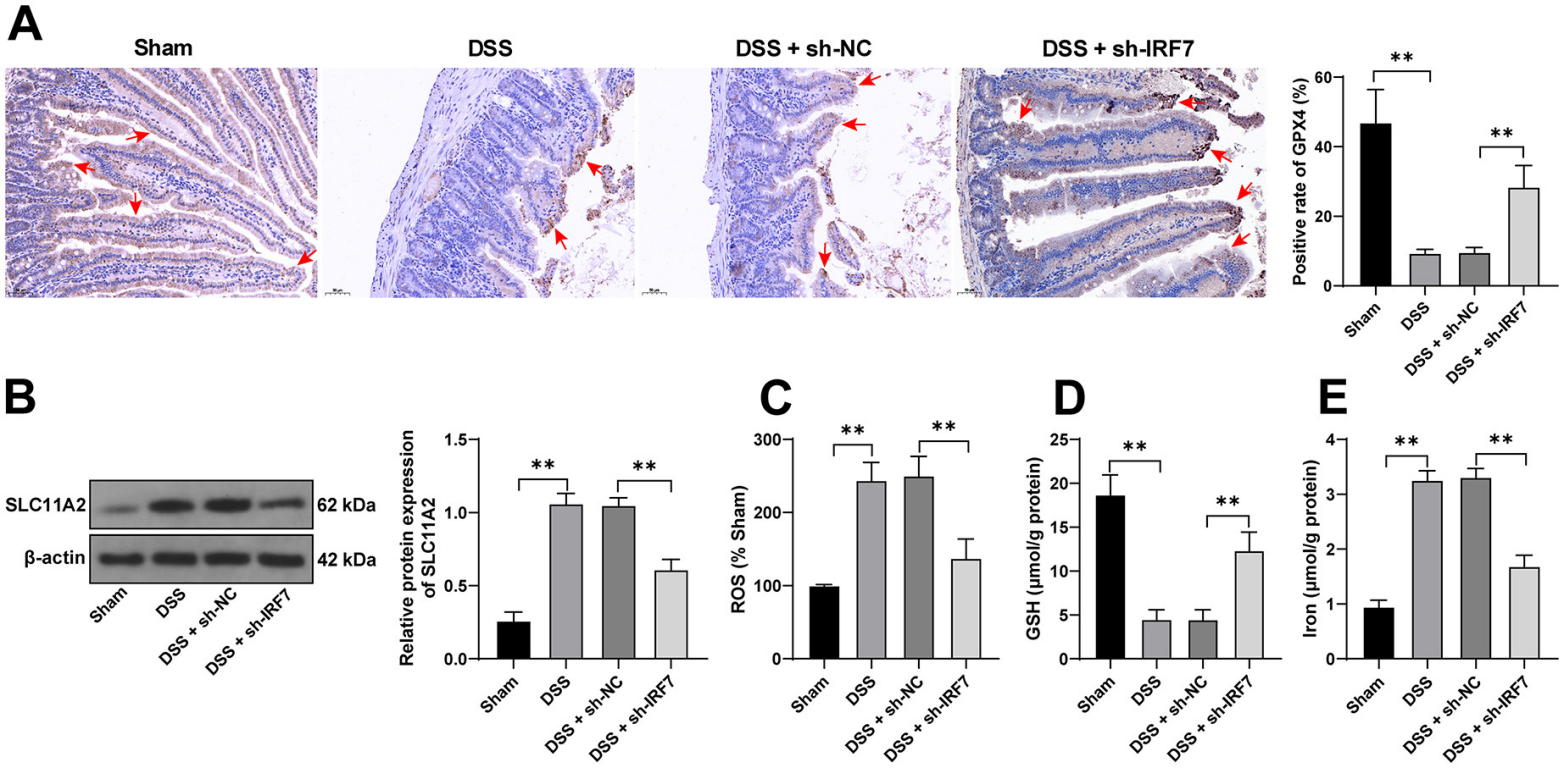
**IRF7 knockdown limits DSS-induced ferroptosis.** (A) The positive rates of GPX4 in mouse colonic tissues were detected using the IHC assay, and the arrows indicated the areas of GPX4 staining; (B) The protein levels of SLC11A2 in mouse colonic tissues were examined using western blotting; (C) The levels of ROS were detected using the fluorescent probe DCFH-DA, the fluorescent intensity of the sham group was 100% and was used to measure the relative fluorescent intensity of the other groups; (D) The levels of GSH in mouse colonic tissues were examined using the commercial kit; (E) The levels of iron ion in mouse colonic tissues were detected using the commercial kit. *n* ═ 6; Comparisons in all panels were assessed by one-way ANOVA; pairwise comparisons after ANOVA analyses were assessed by Tukey’s multiple comparison test; **p < 0.01. IRF7: Interferon regulatory factor 7; UC: Ulcerative colitis; DSS: Dextran sodium sulfate; GPX4: Glutathione peroxidase; IHC: Immunohistochemistry; GSH: Glutathione; ROS: Reactive oxygen species; ANOVA: Analysis of variance.

Ferroptosis has been reported to be associated with intestinal EC death in UC [10]. Ferroptosis levels in the colonic tissues of UC mice were detected, and the results revealed that the positive rates of GPX4 were decreased (*p* < 0.01, Figure 3A), SLC11A2 protein levels were increased (*p* < 0.01, Figure 3B), ROS levels were augmented whereas GSH levels were reduced (*p* < 0.01, Figure 3C and 3D), and iron ion levels were increased (*p* < 0.01, Figure 3E). However, silencing IRF7 averted the above outcomes (*p* < 0.01, Figure 3A–3E), suggesting that the ameliorative effects of IRF7 on DSS-induced UC may be related to IRF7-mediated inhibition of ferroptosis.

### IRF7 knockdown limits ferroptosis of colonic ECs in vitro

**Figure 4.**
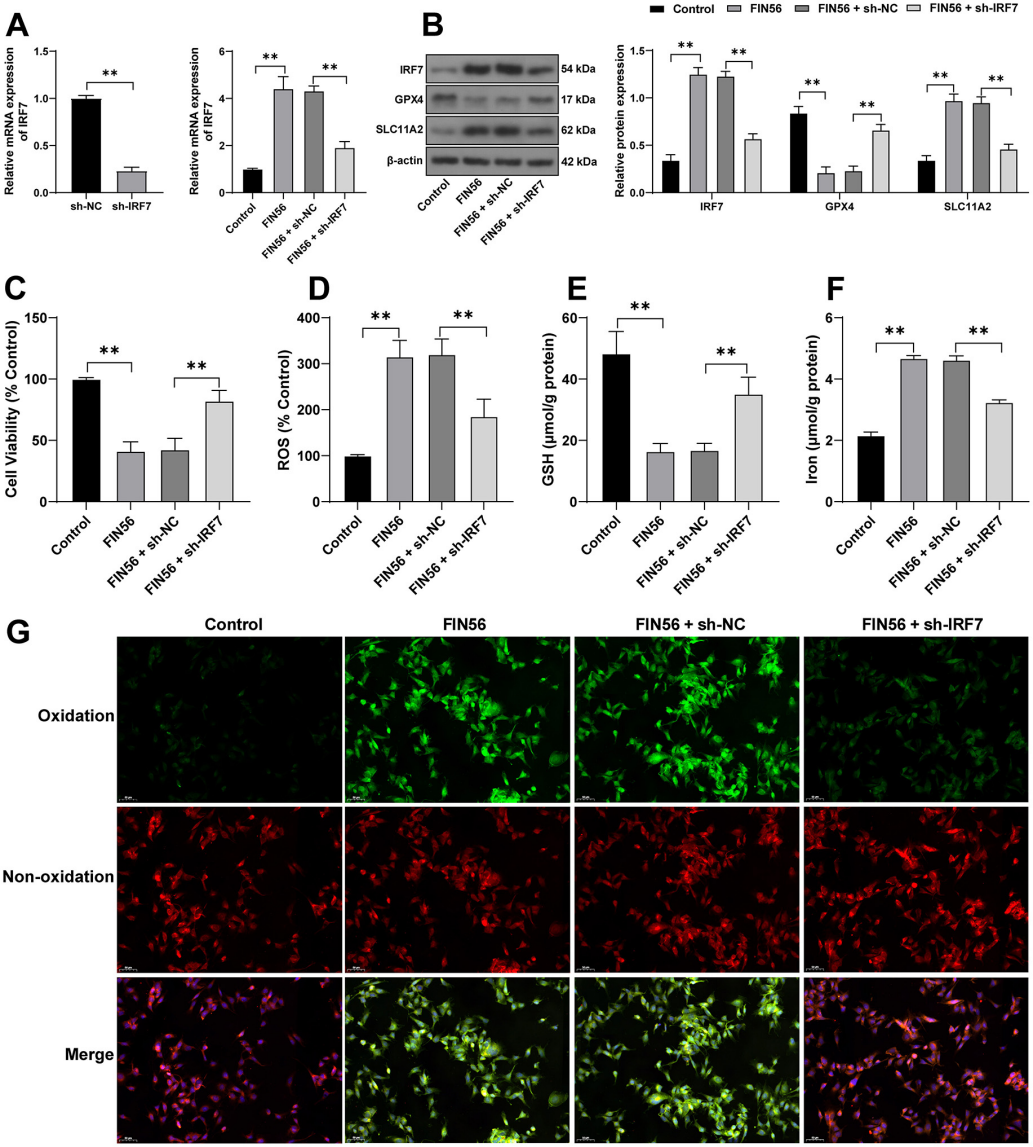
**IRF7 knockdown limits ferroptosis of colonic ECs in vitro.** Primary mouse colonic ECs were isolated and cultured, and then infected with adenovirus-packaged sh-IRF7 and treated with the ferroptosis inducer FIN56 to trigger ferroptosis. (A) The expression levels of IRF7 in ECs were detected using qRT-PCR; (B) The expression levels of IRF7, GPX4, and SLC11A2 in ECs were examined using western blotting; (C) EC viability was measured using the CCK-8 kit; (D) The levels of ROS were detected using the fluorescent probe DCFH-DA, the fluorescent intensity of the control group was 100% and was used to measure the relative fluorescent intensity of the other groups; (E) and (F) The levels of GSH and iron ion in ECs were determined using the commercial kits; (G) The cell lipid peroxidation was detected using the fluorescent probe C11-BODIPY, with the green fluorescence indicating the degree of lipid peroxidation and the red fluorescence indicating non-oxidation. Cell experiments were conducted three times. Comparisons in panels A, C, D, E, and F were assessed by one-way ANOVA; comparisons in panel B were assessed by two-way ANOVA; pairwise comparisons after ANOVA analyses were assessed by Tukey’s multiple comparison test; **p < 0.01. IRF7: Interferon regulatory factor 7; EC: Epithelial cells; GPX4: Glutathione peroxidase GSH: Glutathione; ROS: Reactive oxygen species; qRT-PCR: Quantitative real-time polymerase chain reaction; CCK-8: Cell counting kit 8; ANOVA: Analysis of variance.

To further probe the role of IRF7 in ferroptosis, mouse colonic ECs were infected with sh-IRF7 and the infection efficiency was detected (*p* < 0.01, Figure 4A), and then the infected ECs were treated with FIN56 to construct the ferroptosis cell model. After FIN56 treatment, IRF7 expression levels were elevated (*p* < 0.01, Figure 4A and 4B). Similar to the in vivo experiments, IRF7 expression levels in colonic ECs were decreased by the infection of adenovirus-packaged sh-IRF7 (*p* < 0.01, Figure 4A and 4B), after which the cell viability was increased (*p* < 0.01, Figure 4C), ROS levels diminished, GSH levels increased (*p* < 0.01, Figure 4D and 4E), GPX4 expression levels elevated, SLC11A2 protein expressions decreased (*p* < 0.01, Figure 4B), iron ion levels decreased (*p* < 0.01, Figure 4F), and the cell lipid peroxidation was reduced (Figure 4G). Together, the above results indicated that silencing IRF7 repressed ferroptosis of colonic ECs in vitro.

### IRF7 limits miR-375-3p expression and miR-375-3p inhibits SLC11A2 transcription

**Figure 5.**
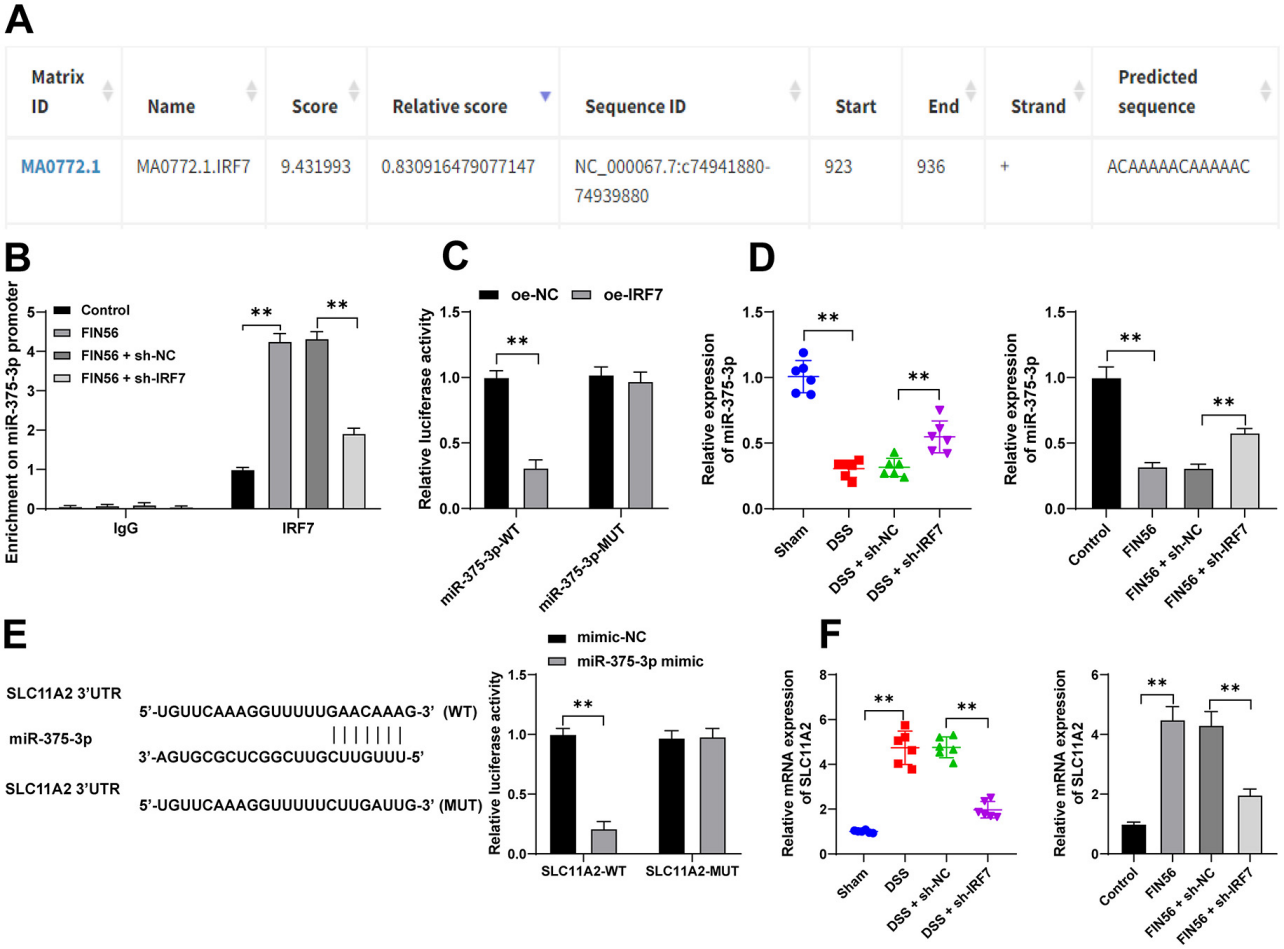
**IRF7 limits miR-375-3p expression and miR-375-3p inhibits SLC11A2 transcription.** (A) The binding sites between IRF7 and the miR-375-3p promoter sequence were predicted by the database JASPAR (http://jaspar.genereg.net/); (B) The enrichment of IRF7 on the miR-375-3p promoter was analyzed using the Ch-IP assay; (C) The binding relationship between IRF7 and the miR-375-3p promoter was verified using the dual-luciferase assay; (D) The expression levels of miR-375-3p in mouse colonic tissues and ECs were detected using qRT-PCR; (E) The binding relationship between miR-375-3p and the SLC11A2 3’UTR was verified using the dual-luciferase assay; (F) The expression levels of SLC11A2 in mouse colonic tissues and ECs were determined using qRT-PCR. *n* ═ 6 (animal experiments); Cell experiments were conducted three times; Comparisons in panels D and F were assessed by one-way ANOVA; comparisons in panels B, C, and E were assessed by two-way ANOVA; pairwise comparisons after ANOVA analyses were assessed by Tukey’s multiple comparison test; **p < 0.01. IRF7: Interferon regulatory factor 7; EC: Epithelial cells; DSS: Dextran sodium sulfate; qRT-PCR: Quantitative real-time polymerase chain reaction; Ch-IP: Chromatin-immunoprecipitation; miR: MicroRNA; ANOVA: Analysis of variance.

Previous study has confirmed the depletion of miR-375 in UC [37]. Here, the binding interaction between IRF7 and the miR-375 promoter sequence was uncovered (Figure 5A). The Ch-IP assay analysis showed that IRF7 was abundantly enriched in the miR-375-3p promoter (*p* < 0.01, Figure 5B) and the dual-luciferase assay revealed that the binding of IRF7 to the miR-375-3p promoter repressed the relative expression of luciferase (*p* < 0.01, Figure 5C). Moreover, miR-375-3p expressions were found to be reduced in UC both in vivo and in vitro and were negatively correlated with IRF7 expression levels (*p* < 0.01, Figure 5D). Additionally, the effect of miR-375-3p on ferroptosis was investigated, and we disclosed that there were targeted binding sites between miR-375-3p and the ferroptosis-related protein SLC11A2 (Figure 5E), and the dual luciferase assay further verified the targeted binding of miR-375-3p to the SLC11A2 3’UTR (*p* < 0.01, Figure 5E). Besides, miR-375-3p expression levels were negatively correlated with SLC11A2 mRNA levels in vivo and in vitro (*p* < 0.01, Figure 5F). Collectively, these data indicated that IRF7 upregulated SLC11A2 transcription by suppressing miR-375-3p expression levels in UC.

### Silencing miR-375-3p averts inhibitory effects of IRF7 knockdown on ferroptosis of colonic ECs

**Figure 6.**
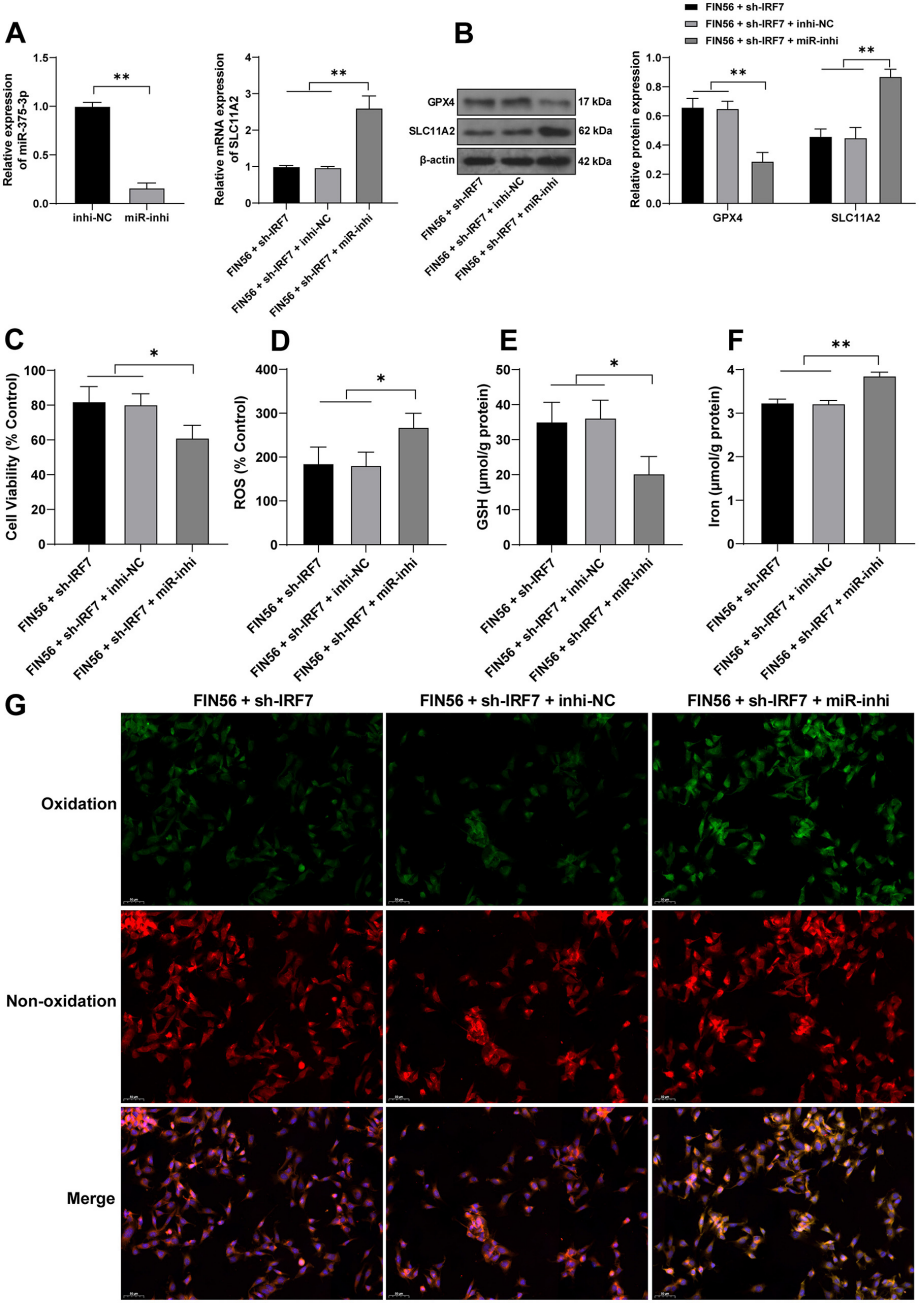
**Silencing miR-375-3p averts inhibitory effects of IRF7 knockdown on ferroptosis of colonic ECs.** Mouse colonic ECs were transfected with miR-375-3p inhibitor (miR-inhi), with inhibitor NC (inhi-NC) as the control, and then the cells were combined with sh-IRF7. (A) The transfection efficiency of miR-375-3p and the mRNA levels of SLC11A2 in the cells after the combined treatment were detected and determined using qRT-PCR; (B) The protein levels of GPX4 and SLC11A2 in the cells were examined using western blotting; (C) Cell viability was measured using the CCK-8 kit; (D) The levels of ROS were detected using the fluorescent probe DCFH-DA, the fluorescent intensity of the control group was 100% and was used to measure the relative fluorescent intensity of the other groups; (E) and (F) The levels of GSH and iron ion in the cells were determined using the commercial kits; (G) The cell lipid peroxidation was detected using the fluorescent probe C11-BODIPY, with the green fluorescence indicating the degree of lipid peroxidation and the red fluorescence indicating non-oxidization). Cell experiments were conducted three times. Comparisons in panel A (left) were assessed by the t-test; comparisons in panels A (right), C, D, E, and F were assessed by one-way ANOVA; comparisons in panel B were assessed by two-way ANOVA; pairwise comparisons after ANOVA analyses were assessed by Tukey’s multiple comparison test; *p < 0.05 and **p < 0.01. IRF7: Interferon regulatory factor 7; EC: Epithelial cells; GPX4: Glutathione peroxidase GSH: Glutathione; ROS: Reactive oxygen species; qRT-PCR: Quantitative real-time polymerase chain reaction; CCK-8: Cell counting kit 8; miR: MicroRNA; ANOVA: Analysis of variance.

At last, mouse colonic ECs were transfected with miR-375-3p inhibitor (miR-inhi) to repress miR-375-3p expression levels (*p* < 0.01, Figure 6A) and combined with sh-IRF7. In the FIN56 + sh-IRF7 + miR-inhi group, both the mRNA and protein levels of SLC11A2 were upregulated (*p* < 0.01, Figure 6A and 6B), and ferroptosis was elevated (*p* < 0.05, Figure 6B–6G). The above outcomes indicated that silencing miR-375-3p neutralized the role of sh-IRF7 in repressing ferroptosis of colonic ECs.

**Figure 7.**
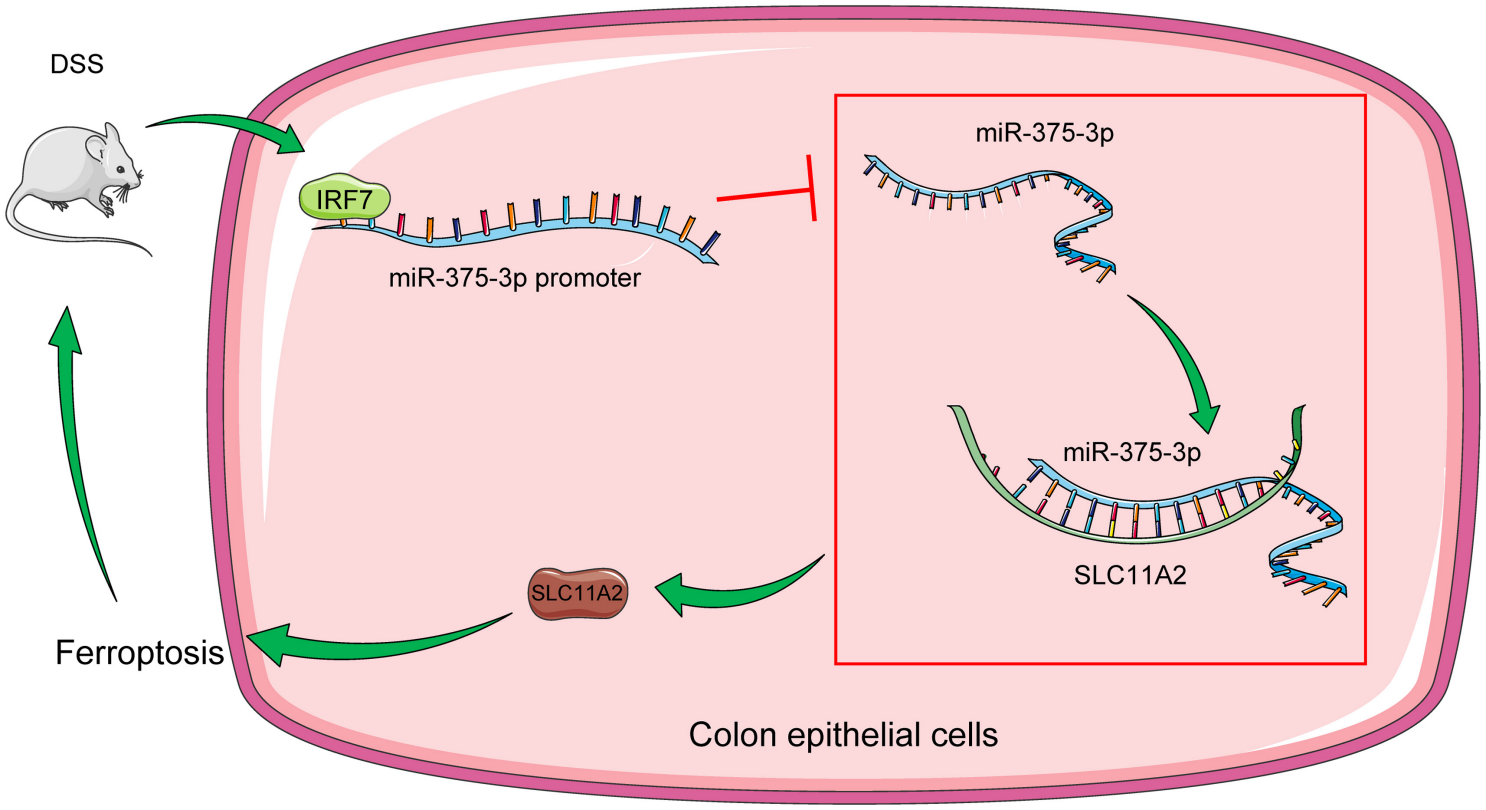
**Molecular mechanism of IRF7 in DSS-induced UC.** DSS treatment increased IRF7 expression levels, and IRF7 binding to the miR-375-3p promoter limited miR-375-3p expression levels and further increased SLC11A2 protein levels by weakening the targeted inhibition of miR-375-3p on SLC11A2, prompting ferroptosis and aggravating UC. IRF7: Interferon regulatory factor 7; UC: Ulcerative colitis; DSS: Dextran sodium sulfate; miRNA: MicroRNA.

## Discussion

UC, which is often diagnosed as an inflammatory disorder of the gastrointestinal system, together with Crohn’s disease constitutes the two clinical subtypes of IBD [38]. Colonic ECs and the epithelium are crucial for maintaining mucosal immunity, and the impairment of the epithelial barrier represents the main manifestation of UC [3, 39]. Ferroptosis is recently identified as a credible hallmark of UC, and the repression of ferroptosis contributes to the rehabilitation of UC and intestinal damages [40]. In this work, our observations uncovered that silencing IRF7 hampered ferroptosis of colonic ECs in UC by the miR-375-3p/SLC11A2 axis (Figure 7).

Mounting studies have highlighted the close association between IRF7 expression levels and inflammation levels in diseases. In systemic sclerosis, silencing IRF7 alleviates dermal fibrosis and inflammatory responses [16]. The depletion of IRF3 and IRF7 weakens diesel exhaust particles-induced pulmonary inflammation by diminishing the releases of IL-1α, IL-6, and IFN-β [41]. In diabetes, IRF7 knockdown increases anti-inflammatory IL-10 production and decreases proinflammatory IL-6 level in bone marrow-derived macrophages [42]. In this study, IRF7 expression levels were upregulated in the colonic tissues of DSS-treated mice. Interestingly, IRF7 depletion alleviated symptoms of UC and histopathological damages in DSS-treated mice and reduced the number of bacterial colonies and the levels of proinflammatory cytokines in vivo. Existing studies have illustrated that the inactivation of the TLRs-TBK1-IRF3/IRF7 pathway by Rebamipide limits inflammation in colonic mucosa of mice with UC [43], and IRF7 overexpression in DCs promotes DSS-triggered intestinal inflammation in mice deficient with lipopolysaccharide-responsive beige-like anchor [18]. Altogether, the above evidence supported the protective role of IRF7 knockdown against DSS-induced UC.

At cellular level, ferroptosis-induced oxidative damage is driven by ROS overproduction, lipid peroxidation, GSH decrease, and GPX4 inactivation [44]. GPX4 is equipped with GSH to prevent cells from ferroptosis by reducing oxidized phospholipids, ROS production, and iron uptake [45, 46]. Notably, the intestinal tissues of IBD patients are evident with the typical signs of ferroptosis [9]. Moreover, the decrease in GPX4 exacerbates ferroptosis in UC as evidenced by reduced GSH and increased malondialdehyde and lactate dehydrogenase [12], and the activation of GPX4 induced by Shaoyao decoction represses ferroptosis in ECs and promotes the repair of barrier function [47]. FIN56 is a potent agent that can induce ferroptosis by increasing GPX4 degradation [48–50]. Among the same type of inducers, the rationale of FIN56 to induce ferroptosis is similar to that of RSL3 [51], but differs from that of Erastin, which initiates ferroptosis by accelerating oxidation by voltage-dependent anion channels [52]. Here, colonic ECs were infected with adenovirus-packaged sh-IRF7 and treated with FIN56. Then, our experimental results showed that silencing IRF7 upregulated the levels of GSH and GPX4 but reduced ROS, SLC11A2, iron ion levels, and lipid peroxidation in DSS-induced mice and FIN56-induced ECs. Upregulation of GSH and GPX4 induced by silencing IRF7 indicated the beneficial effect of silencing IRF7 on preventing ferroptosis. On the other hand, both inflammation and oxidative stress correlated closely with cell ferroptosis [8]. Toll-like receptor 4 mediates the activation of the IRF7/NF-κB pathway to aggravate inflammation in polycystic ovary syndrome [53]. Besides, West Nile virus-infected peripheral blood mononuclear cells represent elevated levels of IFNs and IRF7 and increased oxidative stress [54], and rhinovirus infection facilitates oxidative stress and inflammation with increased IRF7 mRNA levels in chronic obstructive pulmonary disease [55]. Collectively, these data suggested that IRF7 knockdown suppressed ferroptosis in UC in vivo and in vitro.

Existing studies have demonstrated the multifaceted roles of IRF7 in diseases by targeting downstream genes [19, 56]. miR-375-3p upregulation is deemed to reduce the production of IL-1β and IL-6 in ECs in atopic dermatitis [57], and miR-375-3p upregulation in endothelial progenitor cells-isolated extracellular vesicles ameliorates sepsis in rats, as evidenced by alleviated pathological damage and decreased apoptosis, inflammatory responses, and oxidative stress in rat myocardial tissue [58], indicating the beneficial role of miR-375-3p in cell survival. Our data showed that miR-375-3p was underexpressed in UC and IRF7 was able to bind to the miR-375-3p promoter. Additionally, existing studies have elucidated that SLC11A2 activation or upregulation increases ferroptosis in hypoxia/reoxygenation-treated myocardial cells [59], and SLC11A2 knockdown reduces iron deposition and lipid peroxidation and therefore alleviates ferroptosis in rats after subarachnoid hemorrhage [28]. Here, we found that miR-375-3p bound to the SLC11A2 3’UTR, and miR-375-3p expression levels were negatively related to SLC11A2 mRNA levels. Then, miR-375-3p expression levels were decreased in FIN56-treated colonic ECs, after which ferroptosis of these cells was enhanced. miR-375 has been reported to be decreased in the stool of patients with Crohn’s disease [60] and miR-375 depletion increases inflammatory responses in IBD [37]. Furthermore, tetracyclines increase miR-375 expression levels to potentiate mucosal healing and resolution in the context of DSS-included colitis [61]. Altogether, these data indicated that IRF7 limited miR-375-3p expression levels to promote SLC11A2 transcription, and miR-375-3p knockdown reversed the inhibitory effect of silencing IRF7 on ferroptosis of colonic ECs.

Regarding the study limitations, this work is still in the preclinical stage, and there is still a long way to go before the achievement of clinical transformation. We only validated the miR-375-3P/SLC11A2 axis as a downstream of IRF7, without considering other target genes that are potentially regulated by IRF7, and we have yet to probe the IRF7/miR-375-3p/SLC11A2 axis in other cell death processes involved in UC, such as pyroptosis and programmed cell death. Besides, we failed to design alternative shRNAs to perform our study, and therefore there may exist alternative targets that can inhibit IRF7 with higher efficiency and highlight the regulatory role of IRF7 in UC better than the shRNA we used here. In the future, we will analyze our findings based on data from clinical samples, probe other downstream targets, and explore other modes of cell death during UC.

## Conclusion

This work tentatively demonstrated that DSS-induced IRF7 overexpression limited miR-375-3p expression levels by binding to the miR-375-3p promoter and further suppressed miR-375-3p-induced inhibition of SLC11A2 transcription, which accelerated ferroptosis of colonic ECs and exacerbated UC progression. Our study may provide novel targets to facilitate the prevention and treatment of UC.
